# Longitudinal stability of HyperSight^TM^-CBCT based radiomic features in patients with CT guided adaptive SBRT for prostate cancer

**DOI:** 10.1038/s41598-025-99920-x

**Published:** 2025-05-07

**Authors:** Paula Cvachovec, Alicia S. Bicu, Ralf Schmidt, Victor Siefert, Miriam Eckl, Marvin Willam, Sven Clausen, Matthias F. Froelich, Stefan O. Schoenberg, Michael Ehmann, Daniel Buergy, Jens Fleckenstein, Frank A. Giordano, Judit Boda-Heggemann, Constantin Dreher

**Affiliations:** 1 Department of Radiation Oncology, University Medical Centre Mannheim, Medical Faculty Mannheim, University of Heidelberg, Theodor-Kutzer Ufer 1-3, 68167 Mannheim, Germany; 2 DKFZ-Hector Cancer Institute, University Medical Centre Mannheim, Mannheim, Germany; 3 Department of Radiology and Nuclear Medicine, University Medical Centre Mannheim, Medical Faculty Mannheim, University of Heidelberg, Mannheim, Germany; 4 Mannheim Institute for Intelligent Systems in Medicine (MIiSM), Medical Faculty Mannheim, University of Heidelberg, Mannheim, Germany; 5 Junior Research Group ”Intelligent Imaging for adaptive Radiotherapy”, Mannheim Institute for Intelligent Systems in Medicine (MIiSM), University of Heidelberg, Mannheim, Germany

**Keywords:** Radiomics, Adaptive radiotherapy, HyperSight-CBCT, Prostate cancer, Ethos^®^, Prostate cancer, Medical research

## Abstract

CT-guided adaptive radiotherapy (aRT) based on HyperSight^TM^-CBCT provides high-quality imaging, allowing quantitative radiomic feature analysis as a monitoring tool. This study comprehensively evaluates the stability of radiomic features, as potential imaging biomarkers, in pelvic structures of prostate cancer patients treated with adaptive stereotactic body radiation therapy (SBRT). Between December 2023 and July 2024, 32 patients with localized prostate cancer underwent adaptive SBRT at the Ethos^®^ linear accelerator (Varian, Siemens Healthineers) with HyperSight-CBCT imaging. Longitudinal stability was assessed by intraclass correlation coefficient (ICC) over five fractions of aRT for target structures and non-hollow organs at risk. In pooled organs at risk, 93.0% of features showed very high stability (ICC > 0.9) compared to 67.4% in pooled target structures, indicating significantly lower stability for target structures (*p* = 0.00009129). Second-order features demonstrated greater stability than conventional and shape-based features (*p* = 0.0433, *p* = 0.0252). Fraction number significantly affected longitudinal prostate feature variability (*p* = 0.0135). This study comprehensively analyzed HyperSight-CBCT imaging to evaluate longitudinal stability of radiomic features during adaptive SBRT for prostate cancer. The trends observed will provide a framework for future CT-guided aRT studies, facilitating quantitative imaging analysis of radiological biomarkers for clinical translation and improving personalized treatment.

## Introduction

Adaptive radiotherapy (aRT), a closed-loop process that adjusts radiation treatment to the patient’s daily anatomy, was first introduced by Yan et al.^1^. The integration of advanced imaging technologies has further refined aRT approaches, facilitating precise treatment adaptation and contributing to improved patient outcomes^2–5^. Consequently, aRT is particularly relevant in ultra-hypofractionated stereotactic body radiation therapy (SBRT), which delivers high doses per fraction, and in the pelvic region, an area prone to interfractional changes caused by fluctuating rectal and bladder volumes^6,7^.

The HyperSight-CBCT imaging unit, available on both the Ethos^®^ and Halcyon platforms, and more recently on the TrueBeam platform (Varian, Siemens Healthineers), represents a significant advancement in CBCT imaging by providing diagnostic-grade image quality through advanced software solutions and hardware integration^8–10^. This advancement supports optimized aRT strategies with treatment intensification and enables qualitative and quantitative monitoring of tissue changes^11–14^. Quantitative imaging parameters derived from this system could provide valuable insights into tumor response and normal tissue reactions. While metrics such as the mean and maximum volumetric values of the volumes of interest (VOI) are already part of routine diagnostic evaluations in oncologic radiology^15^, radiomics analyses significantly enhance VOI assessments by offering computerized mathematical imaging features that characterize morphology and tissue heterogeneity, allowing for more precise lesion and tissue characterization^16–18^.

The combination of high-precision aRT and advanced quantitative imaging techniques, such as radiomics analyses, has the potential to revolutionize monitoring strategies by moving beyond isolated qualitative evaluations of visible tissue changes^19^. However, routine CBCT scans are usually unsuitable for quantitative imaging analyses due to pronounced artifacts, reduced image quality, and consequently challenges in accurately identifying VOIs^20–23^. For instance, compared to radiomic features derived from diagnostic computed tomography (CT) scans, the predictive value of CBCT-based radiomic features in patients undergoing radiotherapy for lung cancer remains unclear^24–26^. Preliminary findings on the clinical application of CBCT-based radiomics in patients receiving standard image-guided radiotherapy (IGRT) for prostate cancer suggest that radiomics analysis of daily standard CBCT scans may enhance the prediction of treatment toxicity and response^27,28^.

Although routine CBCT-based parameters for prostate cancer show promise, they cannot yet be standardized for reliable monitoring. Radiomics analysis using the advanced HyperSight CBCT imaging mode holds potential to further enhance the clinical utility of CBCT-based parameters, particularly for patients undergoing advanced, high-precision RT approaches such as adaptive SBRT. Therefore, this study aims to comprehensively investigate the longitudinal stability of HyperSight-CBCT-derived radiomic features in pelvic structures of prostate cancer patients treated with adaptive SBRT.

## Material and methods

### Study design and patients

A total of 32 prostate cancer patients (Table 1) undergoing adaptive radiotherapy by means of stereotactic body radiotherapy (SBRT) were prospectively enrolled in a registry trial set up for aRT at the Medical Faculty Mannheim, University Heidelberg. Ethical approval for this study was obtained from the Institutional Ethics Committee (reference number 2023-557), and all procedures were conducted as per relevant guidelines and regulations. All patients were treated between December 2023 and July 2024 and informed consent was obtained from all the participants. Additional Androgen Deprivation Therapy (ADT) was administered based on the physician’s decision and shared decision making in dependency on the individual risk factors. During adaptive radiotherapy, one patient started concurrent ADT. Additionally, three femora with total hip replacements were excluded from the statistical analysis. Detailed patient characteristics are summarized in Table 1.

**Table 1:** Patient characteristics.

Characteristics	n (%)
Patients	32 (100%)
Age (years)	
Mean ± SD	69.8 ± 7.4
Gleason score	
6	5 (15.63%)
7 (3 + 4)	23 (71.88%)
7 (4 + 3)	3 (9.37%)
8	1 (3.12%)
Tumor stage	
cT1	29 (90.62%)
cT2	3 (9.38%)
Initial PSA[ng/ml]	
Mean ± SD	8.2 ± 5.4

PSA (prostate-specific antigen), SD (standard deviation).

### Treatment

**Fig. 1:**
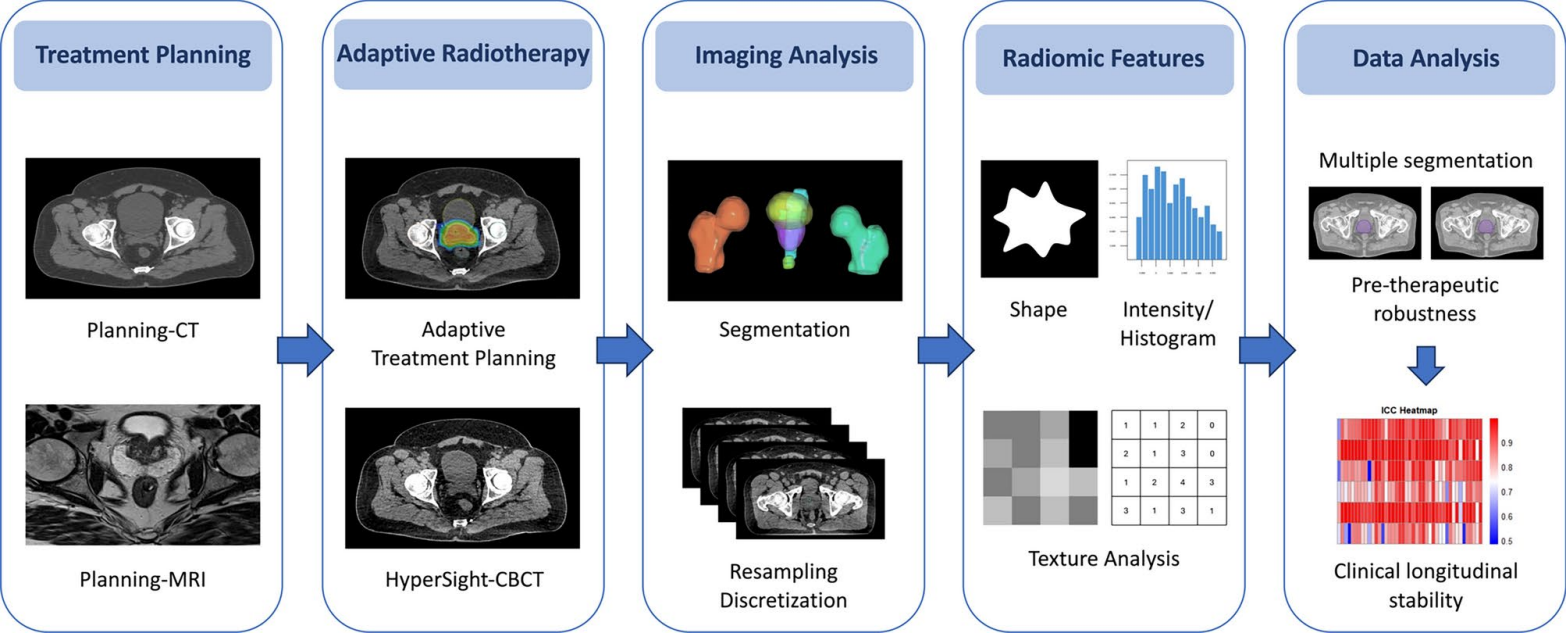
Workflow of adaptive radiotherapy in patients with prostate cancer undergoing ultra-hypofractionated stereotactic body radiotherapy. Target delineation is based on pelvic CT (computed tomography) and MRI (magnetic resonance imaging) scans which are used for treatment planning. Adaptive radiotherapy (aRT) is performed based on fractional HyperSight-CBCT (conebeam computed-tomography) scans, with online adaptive segmentation of normal tissue organs and target volumes. Following fractional aRT treatment, HyperSight-CBCT image processing is conducted, and radiomic features (e.g. of shape, intensity/histogram and texture analysis) are extracted using the open-source software LIFEx (version 5.10, https://www.lifexsoft.org)^30^. Subsequently, pre-therapeutic robustness, based on multiple segmentations and clinical longitudinal stability analyses across five fractions of adaptive radiotherapy are performed.

Adaptive radiotherapy was applied using the ring-based Ethos^®^ linear accelerator (Varian, Siemens Healthineers). Treatment planning utilized the Ethos^®^ Appliance System and was based on a planning CT scan (Brilliance BigBore, Philips, Netherlands) and multiparametric magnetic resonance imaging (conducted at different diagnostic centers) of the prostate and pelvic organs (Fig. 1). The clinical target volume (CTV) was defined to include the prostate and the proximal 1 cm of seminal vesicles with an additional margin of 2 mm. The CTV was expanded by 2–4 mm to form the planning target volume (PTV). The prescribed doses were 40 Gy to the CTV and 36.25 Gy to the PTV, both delivered in 5 fractions. For patients with a Gleason Score of 7(4 + 3) or 8(4 + 4), a second PTV encompassing the prostate and the proximal 2 cm of seminal vesicles with an additional margin of 4–8 mm was prescribed a dose of 30 Gy in 5 fractions. Margin adjustments were permitted based on clinical decision. The institutional radiation oncology criteria and planning parameters were derived from the PACE trial^29^. Online adaptive contouring of normal tissue organs and target volumes on HyperSight-CBCT scans was performed by a board-certified, experienced radiation oncologist. Treatments were delivered daily or on alternate days over a period of 1–2 weeks.

### HyperSight-CBCT imaging

Fractional HyperSight-CBCT imaging prior to aRT was performed using the preset “Pelvis” and the “iCBCT” reconstruction mode, with additional metal artefact reduction (MAR) in presence of pelvic implants (three total hip replacements and one femoral nail). It should be noted that the CBCT mode “iCBCT Acuros algorithm” was not employed. As the iCBCT algorithm was executed on a HyperSight equipped device, the CBCT scans analyzed in this trial are referred to as HyperSight-CBCT. The specific characteristics of the HyperSight-CBCT “Pelvis” protocol are as follows: an energy of 125 kV, an exposure of 469 ± 1 mAs, a scan duration of 5.9 s and a reconstructed slice thickness of 2 mm. For each fraction, HyperSight-CBCT imaging prior to aRT consisted of two acquisitions: one for initial adaptive treatment planning and one immediately before dose delivery to ensure accurate dose application.

### Imaging analysis and radiomic features extraction

The HyperSight-CBCT scans for aRT were extracted from the Ethos^®^ Appliance System for each patient and fraction using the Velocity software (Varian, Siemens Healthineers) (Fig. 1). From these HyperSight-CBCT scans, the clinically semi-automatically segmented AI(Artificial Intelligence)-based (and reviewed/adapted by a board-certified radiation oncologist) non-hollow target structures (prostate and seminal vesicles) and non-hollow organs at risk (femoral head and neck on both sides and penile bulb) from the Ethos^®^ aRT workflow were included in the analysis. Secondary segmentations to assess robustness through multiple delineations were also performed, as described in the following sections. To ensure robustness, variable hollow organs at risk of online aRT (rectum, bladder, and bowel) were excluded from analysis.

Whole-VOI segmentations from the HyperSight-CBCT scans were analyzed using the open-source software LIFEx (version 5.10, https://www.lifexsoft.org) which provided a graphical user interface (GUI) for image processing and radiomic feature extraction^30^. Standardized preprocessing steps included spatial resampling to 1 × 1 × 1 mm^3^, intensity discretization with 400 grey levels, and absolute intensity rescaling within a range of −1000 – 3000 Hounsfield units (HU). A total of 43 radiomic features were calculated for analysis, comprising first-order conventional values (CONVENTIONAL) and shape matrix (SHAPE) features as well as second-order grey-level co-occurrence matrix (GLCM), grey-level run length matrix (GLRLM), neighboring grey-level dependence matrix (NGLDM), and grey-level zone length matrix (GLZLM) features.

This study was conducted in two parts.

Part A: Pre-therapeutic robustness of radiomics analysis.

To comprehensively assess the impact of contouring variability and image acquisition on the robustness of radiomic features, independent of dose-related effects, a baseline analysis for the exemplary VOI prostate was performed on the first 10 patients using iCBCT-based HyperSight-CBCT imaging prior to the first fraction. Radiomic feature robustness was assessed through: (1) test–retest analysis based on the two HyperSight-CBCT scans acquired prior to the first fraction, with additional segmentation performed by a board-certified radiation oncologist on the second HyperSight-CBCT scan; (2) intra-reader analysis using the first HyperSight-CBCT scan acquired prior to the first fraction, with additional segmentation by the same board-certified radiation oncologist; (3) inter-reader analysis using the first HyperSight-CBCT scan acquired prior to the first fraction, with additional segmentation by a second board-certified radiation oncologist; and (4) comparison of these results with each other, as well as with the clinical longitudinal stability across five fractions, as described in part B.

Pre-therapeutic robustness was evaluated by extracting 43 radiomic features and assessing the intraclass correlation coefficient (ICC).

Part B: Clinical longitudinal stability of radiomic features across five fractions of aRT.

A total of 160 HyperSight-CBCT scans from the complete cohort of 32 patients were analyzed, encompassing prostate, seminal vesicles, bilateral femoral head and neck, and penile bulb as VOIs from the initial HyperSight-CBCT scans of each of the five fractions. For each VOI, 43 radiomic features were extracted, and their longitudinal stability across five fractions was assessed using the ICC.

### Statistical analysis

Statistical analyses were performed using R (version 4.2.1, R Foundation for Statistical Computing, Vienna, Austria). Intraclass correlation coefficients (ICCs) were calculated using a two-way mixed model for absolute agreement to assess the robustness of radiomic features in the pre-therapeutic setting (intra-reader, inter-reader, test–retest robustness analysis for the prostate) as well as for clinical application across five fractions in different VOIs (prostate, seminal vesicle, pooled left and right femoral head/neck[femora], and penile bulb). The ICC values were categorized as very high (> 0.9), high (0.75–0.9), moderate (0.5–0.75), or low (< 0.5) stability^31^. Differences in pre-therapeutic robustness measures, as well as ICC differences across VOIs in the clinical application setting, were assessed using the Friedman test with radiomic features as grouping factor. Post-hoc comparisons were performed using the Conover and Dunn’s tests (Bonferroni-Holm/Bonferroni-corrected). The Wilcoxon signed-rank test was used to compare ICCs between pooled target structures and organs at risk.

To analyze the influence of radiomic feature groups (conventional, shape-based, and second-order features) on ICC values, a Kruskal–Wallis test was applied, followed by pairwise Dunn’s tests (Bonferroni-corrected). Longitudinal changes in radiomic feature values were examined as differences from fraction 1 using linear regression, with the initial structure volume as a covariate and fraction number, VOI, and their interaction as independent variables. Statistical significance was defined as *p* < 0.05 for all tests.

## Results

### Pre-therapeutic robustness of radiomic features

Intra-reader (median ICC: 0.990 ± 0.034 IQR (interquartile range)), inter-reader (median ICC: 0.994 ± 0.017 IQR) and test–retest (median ICC: 0.965 ± 0.086 IQR) analyses demonstrated very high, pre-therapeutic robustness of the radiomic features for the prostate as a baseline prior to aRT dose application. The pre-therapeutic robustness, as assessed by intra-reader (*p* = 2.58·10^−12^), inter-reader (*p* = 2.97·10^−21^), and test–retest analyses (*p* = 0.032), was significantly higher compared to longitudinal 5-fractions-stability (ICC median: 0.870 ± 0.186 IQR).

### Clinical longitudinal stability of radiomic features

#### Longitudinal stability of radiomic features across five fractions of aRT

**Fig. 2:**
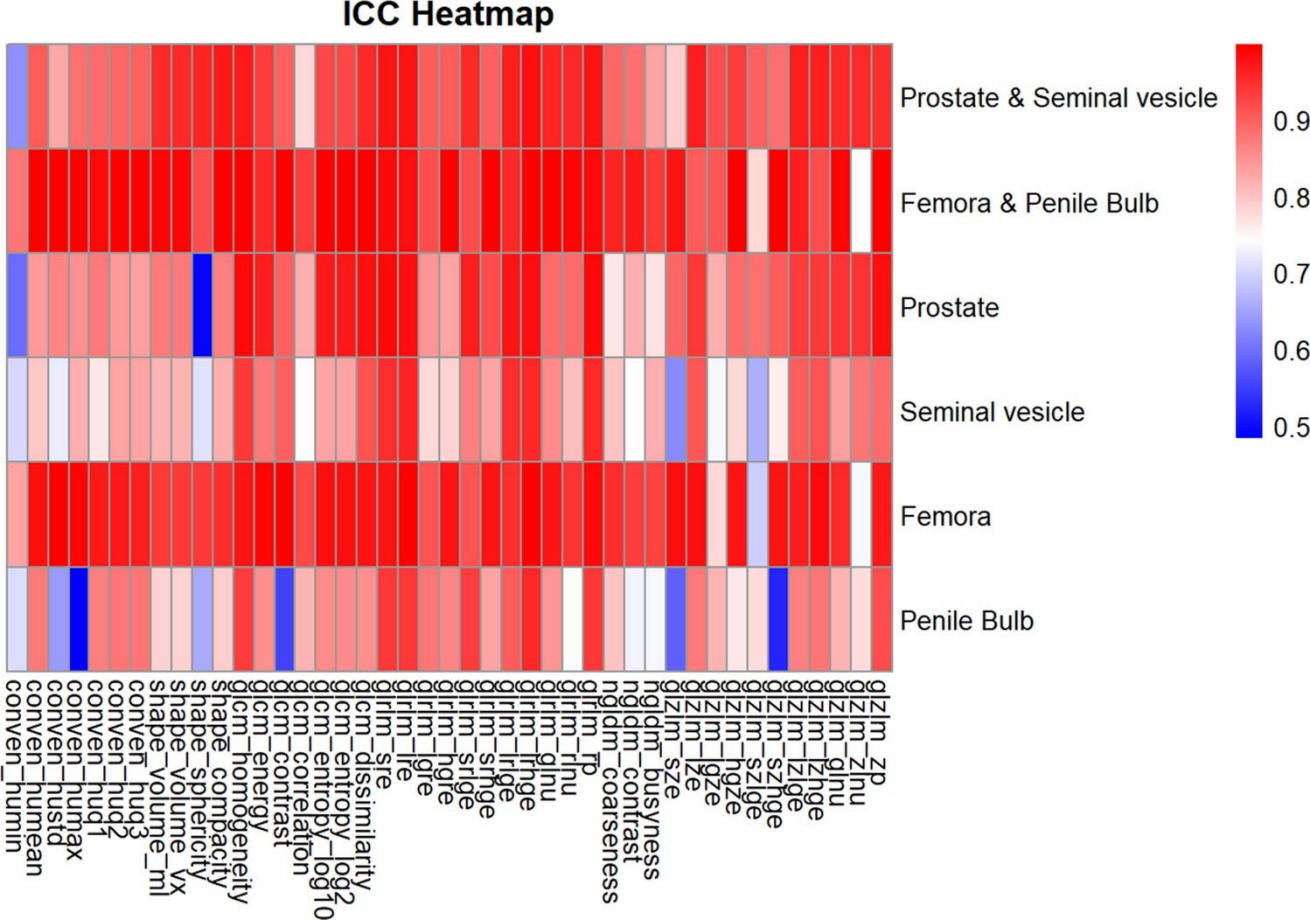
Heatmap displaying the intraclass correlation coefficient (ICC) of radiomic features in different volumes of interest (VOIs) in fractional HyperSight-CBCT scans during adaptive SBRT of prostate cancer: Prostate & Seminal vesicle (pooled), Femora & Penile Bulb (pooled), Prostate, Seminal vesicle, Femora (pooled left &right) and Penile Bulb. Colors ranging from dark red (ICC close to 1.0, indicating very high stability) to blue (ICC near 0.5, indicating low stability). Radiomic features: conventional values (CONVEN), shape-based features (SHAPE), grey-level co-occurrence matrix (GLCM), grey-level run length matrix (GLRLM), neighboring grey-level dependence matrix (NGLDM), and grey-level zone length matrix (GLZLM).

ICC values of the prostate varied the most, reflecting lower stability in shape features and conventional features, yet very high stability in texture features such as glcm_homogeneity (Fig. 2). Similarly, seminal vesicles displayed the greatest stability in glcm_homogeneity, though stability in conventional features was more moderate. For the pooled femora, ICC values remained consistently high, while ICC values for the penile bulb exhibited greater variability. In general, organs at risk demonstrated higher stability compared to target structures. Table 2 lists the three radiomic features with the highest and lowest ICC values for each VOI and VOI group.

**Table 2:** Summary of the three radiomic features with the highest and lowest intraclass correlation coefficients.

VOI	3 Radiomic features with highest ICC	3 Radiomic features with lowest ICC
Femora & Penile bulb	conven_hustd (0.998), shape_compacity (0.997), glcm_dissimilarity (0.997)	glzlm_zlnu (0.742), glzlm_szlge (0.777), glrlm_lgre (0.921)
Femora	conven_hustd (0.996), glrlm_lrhge (0.996), glrlm_lre (0.994)	glzlm_szlge (0.693), glzlm_zlnu (0.735), glzlm_lgze (0.779)
Penile bulb	glrlm_lrhge (0.954), glrlm_rp (0.941), glrlm_sre (0.939)	conven_humax (0.484), glzlm_szhge (0.524), glzlm_sze (0.584)
Prostate & Seminal vesicle	glrlm_lrhge (0.978), glrlm_lre (0.978), glrlm_rp (0.977)	conven_humin (0.631), glcm_correlation (0.782), glzlm_sze (0.787)
Prostate	glcm_homogeneity (0.985), glrlm_rp (0.984), glrlm_sre (0.983)	shape_sphericity (0.491), conven_humin (0.597), glcm_correlation (0.823)
Seminal vesicle	glrlm_lre (0.961), glrlm_rp (0.955), glrlm_lrhge (0.954)	glzlm_sze (0.623), glzlm_szlge (0.661), conven_humean (0.795)

Intraclass correlation coefficients (ICC) of radiomic features for different volumes of interest (VOI) in fractional HyperSight-CBCT scans during adaptive SBRT of prostate cancer. ICC values in brackets. Radiomic features: conventional values (CONVEN), shape-based features (SHAPE), grey-level co-occurrence matrix (GLCM), grey-level run length matrix (GLRLM), and grey-level zone length matrix (GLZLM).

**Fig. 3:**
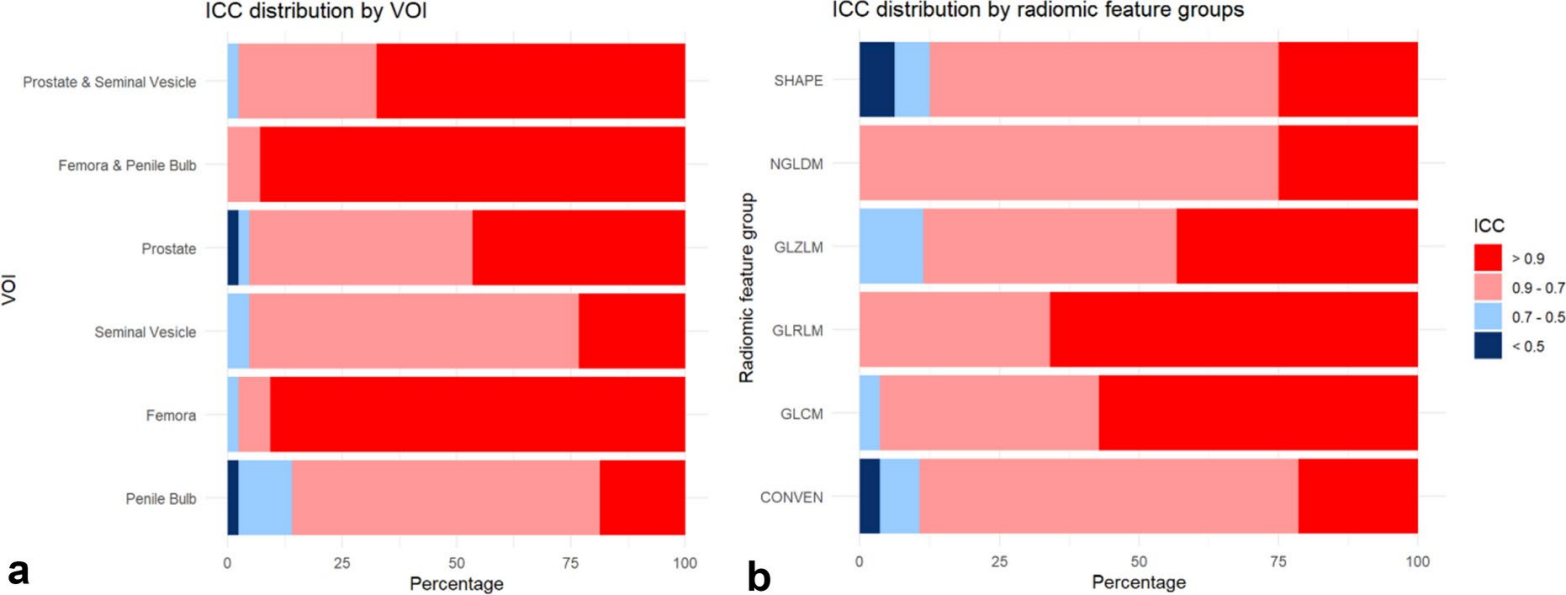
Intraclass correlation coefficient of radiomic features in fractional HyperSight-CBCT scans during adaptive SBRT of prostate cancer: (**a**) Bar plot illustrating the percentage of radiomic features with very high (ICC > 0.9), high (ICC = 0.75–0.9), moderate (ICC = 0.5–0.75), and low (ICC < 0.5) stability across different volumes of interest. (**b**) Bar plot showing the distribution of ICCs of radiomic feature groups (conventional values (CONVEN), shape matrix (SHAPE), grey-level co-occurrence matrix (GLCM), grey-level run length matrix (GLRLM), neighboring grey-level dependence matrix (NGLDM), and grey-level zone length matrix (GLZLM)) across all volumes of interest (VOIs).

Among pooled target structures, 67.4% of the radiomic features demonstrated very high stability (ICC > 0.9), while 30.2% exhibited high stability (ICC 0.7–0.9) (Fig. 3A). In contrast, the pooled organs at risk showed a greater proportion of radiomic features with very high stability, with 93.0% of features having ICCs greater than 0.9 and only 7.0% falling within the 0.7–0.9 range (Fig. 3A).

When considering the stability of all 43 extracted radiomic features across different feature groups, notable variations were observed. GLRLM and GLCM features demonstrated the highest proportions of very high stability (65.9% and 57.1%, respectively) (Fig. 3B). In comparison, conventional features exhibited lower stability, with 67.9% of features classified within the high stability range (ICC 0.7–0.9) (Fig. 3B).

#### Clinical organ-specific stability across five fractions of aRT

**Fig. 4:**
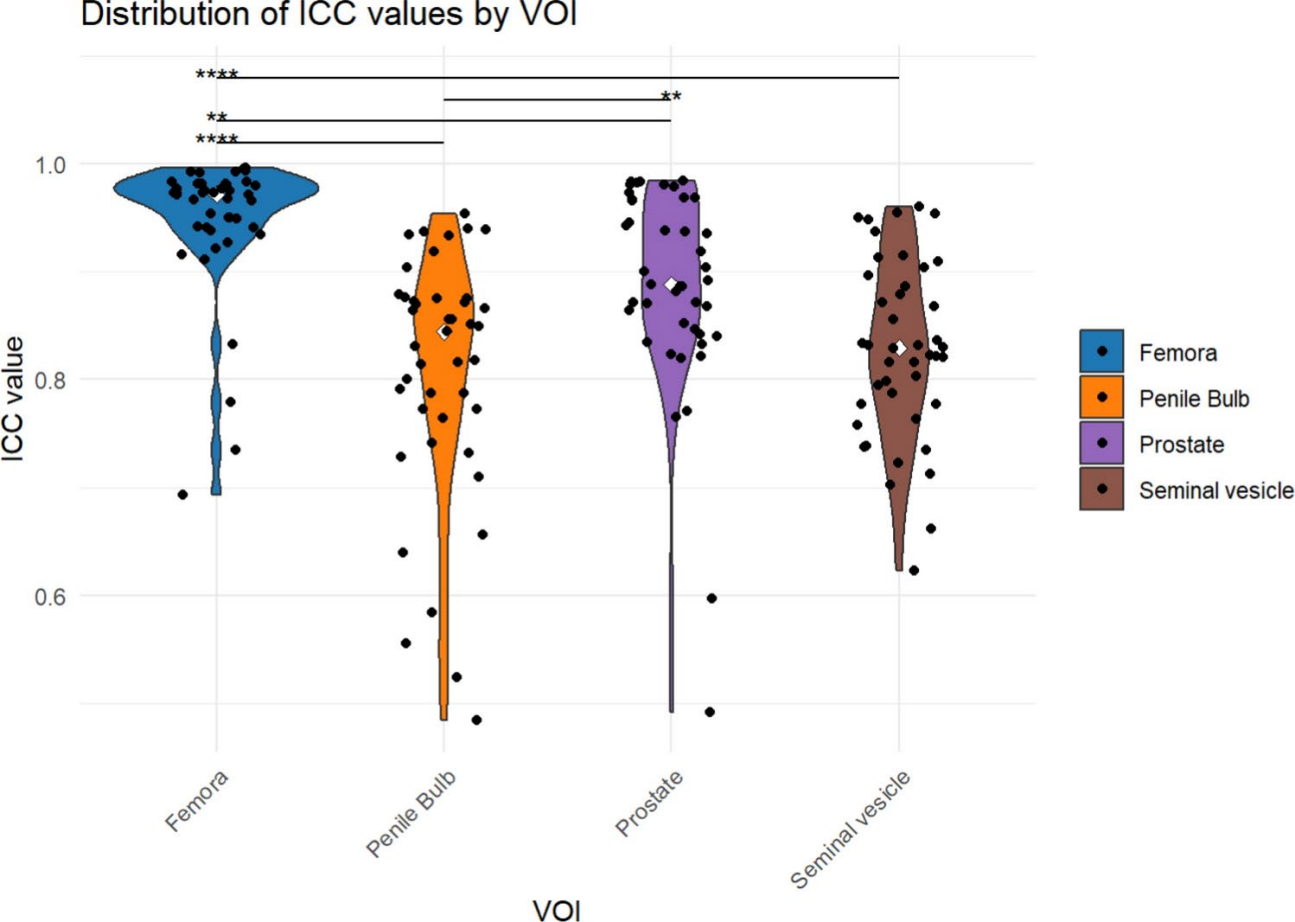
Intraclass correlation coefficient (ICC) of radiomic features in fractional HyperSight-CBCT scans during adaptive SBRT of prostate cancer: Violin plots showing the distribution of ICC values for each volume of interest (VOI). Black points represent individual ICCs for each radiomic feature within each VOI. White points represent the median ICC values. Significant differences between VOIs, as determined by pairwise comparisons, are indicated by * *p* ≤ 0.05, ** *p* ≤ 0.01, and **** *p* ≤ 0.0001.

A significant impact of the specific VOI on the ICC values was identified (*p* = 6.921·10^−16^) with radiomic features considered as a grouping factor. Subsequent pairwise comparisons identified significant differences in ICC values between individual VOIs (Fig. 4). Furthermore, comparison of the pooled target structures (median ICC: 0.933 ± 0.063 IQR (Interquartile range)) with the pooled organs at risk (median ICC: 0.993 ± 0.05 IQR) revealed a significant reduction in ICC values for target structures compared to organs at risk (*p* = 9.129·10^−5^).

#### Impact of radiomic feature group on treatment-based stability across five fractions of aRT

Further analysis of radiomic feature groups, with ICC values for NGLDM, GLZLM, GLCM, and GLRLM categorized as second-order texture features, revealed a significant influence on ICC values (*p* = 0.0101). The median ICC values ± IQR were 0.841 ± 0.089 for conventional features, 0.904 ± 0.139 for second-order features, and 0.819 ± 0.101 for shape-based features.

**Fig. 5:**
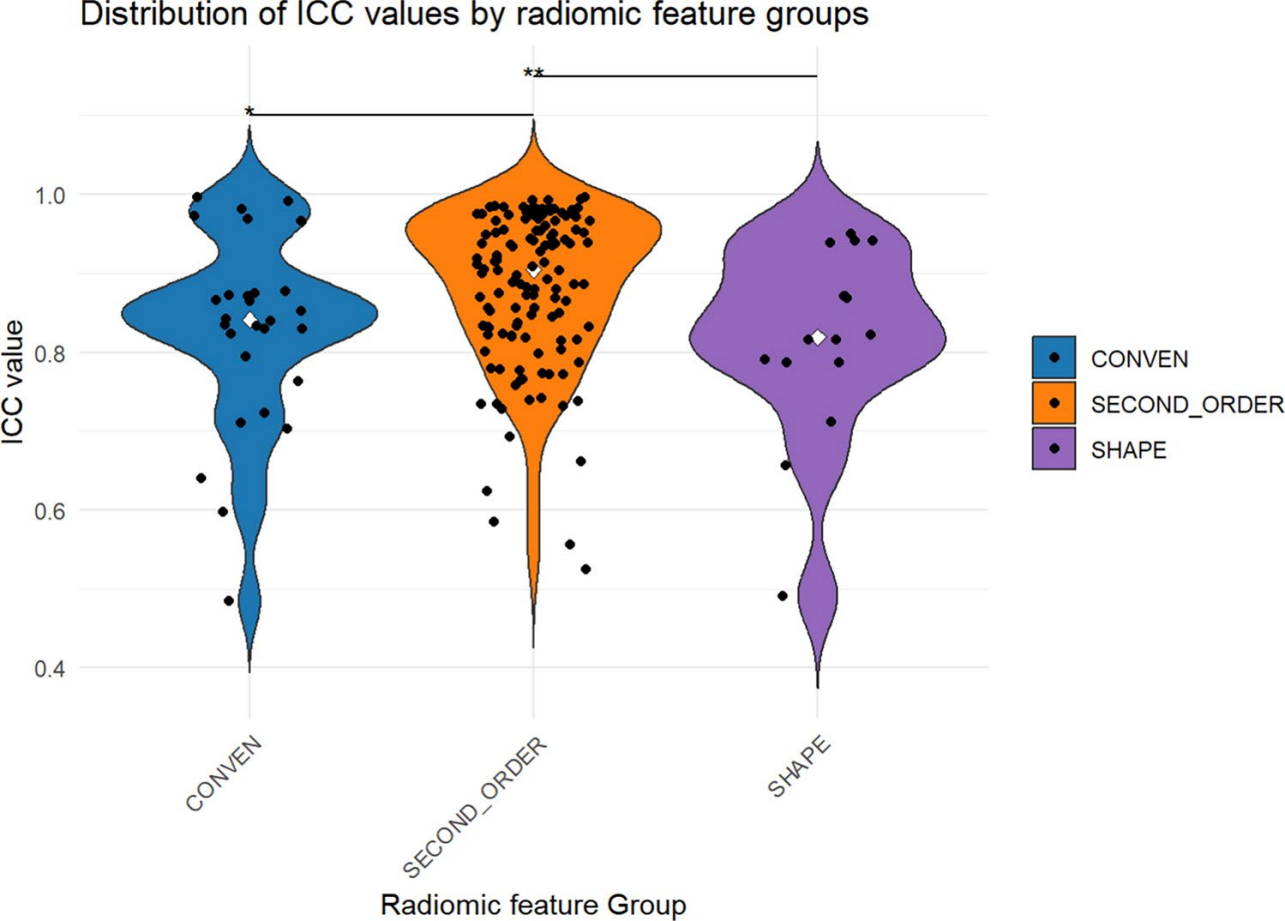
Intraclass correlation coefficient (ICC) of radiomic features in fractional HyperSight-CBCT scans during adaptive SBRT of prostate cancer: Violin plots showing the distribution of ICCs in different radiomic feature groups: conventional (CONVEN), second-order (NGLDM, GLZLM, GLCM, GLRLM), shape-based features (SHAPE). Black points represent individual ICCs for each radiomic feature. White points indicate median. Significant differences between feature groups are marked with * *p* ≤ 0.05 and ** *p* ≤ 0.01.

Dunn’s post-hoc comparisons revealed significantly decreased values for conventional features compared to second-order features (*p* = 0.0433), and for shape-based features compared to second-order features (*p* = 0.0252) (Fig. 5).

#### Longitudinal course of radiomic features during five fractions of aRT

**Fig. 6:**
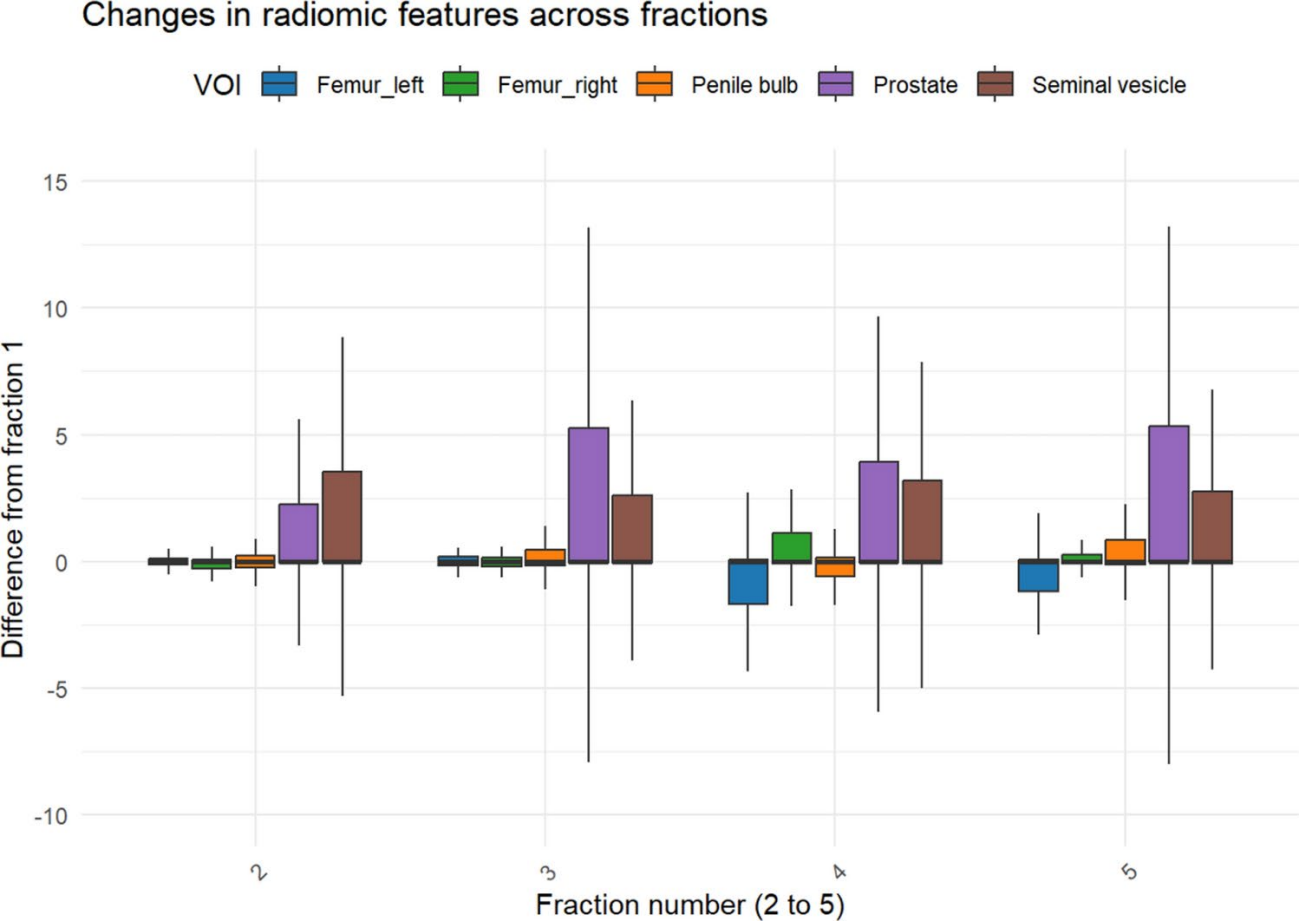
Boxplots showing the distribution of differences in radiomic features in fractional HyperSight-CBCT scans between fractions 2–5 and fraction 1 during adaptive SBRT of prostate cancer: Colored boxes denote the distribution of differences for each volume of interest (VOI). Black lines represent the median, box edges denote the interquartile range (IQR), whiskers represent the range within 1.5 times the IQR. A pronounced difference can be seen longitudinally for the VOI prostate.

Regression analysis of radiomic feature differences across fractions 2–5, relative to fraction 1, revealed a significant interaction between fraction number and the VOI prostate (*p* = 0.0135) (Fig. 6). This finding suggests an increasing variability in prostate radiomic features with higher fraction numbers and cumulative dose application to the prostate. For other VOI, no significant increases in differences were observed. The initial structure volume had no significant effect on inter-fractional radiomic changes.

## Discussion

We here provide the first comprehensive quantitative analyses of pre-therapeutic robustness and longitudinal stability of HyperSight-CBCT-based radiomic features in patients undergoing adaptive SBRT for prostate cancer. Remarkably, the analysis demonstrated very high pre-therapeutic robustness of radiomic features in the prostate and revealed a distinct pattern, with the longitudinal stability of radiomic features significantly decreasing as dose exposure to the target structure escalated—an insight not observed in other studied organs. Overall, conventional and shape-based features demonstrated significantly lower stability compared to second-order radiomic features throughout the longitudinal course of adaptive SBRT.

Radiomics analysis of medical imaging has become a focal point of clinical research, particularly in oncologic radiology and radiation oncology. This research is driven by the potential of radiomics to revolutionize personalized medicine and support decision-making, particularly through its use as imaging biomarkers^16,18^. However, regarding prostate cancer, radiomics studies have primarily focused on MRI-based analyses^32–34^. Investigations by Tanadini-Lang et al., Osman et al., and Ching et al. have demonstrated the predictive value of CT-based radiomic features for risk stratification in prostate cancer^35–37^. These findings suggest that CT-based imaging can provide valuable insights into various oncological risk stages of prostate cancer, highlighting the untapped potential of longitudinal CT monitoring through daily CBCT imaging during radiotherapy for advanced response assessment. Despite its potential, the current understanding of CBCT-based radiomics analysis as part of IGRT in daily clinical practice remains limited^34,38^. Standard CBCT imaging is typically associated with significantly reduced imaging quality and potential artefacts in relevant regions of interest, challenging the reliability of quantitative radiomic parameter analysis^20–23^. While standard CBCT scans are primarily used for visual mapping of the main anatomical structures during IGRT, their value for advanced quantitative imaging analysis—capable of capturing therapeutic changes beyond what is visually discernible—remains poorly understood and limited due to the inferior image quality. Encouragingly, Delgadillo et al. demonstrated a high repeatability of CBCT based radiomic features using the iCBCT reconstruction mode in prostate cancer radiotherapy patients^39^. However, unlike the current study, their analysis did not examine the longitudinal course of radiomic features or utilize HyperSight-CBCT-based imaging at an O-ring linear accelerator. In a separate trial, Delgadillo et al. explored the potential of quantitative delta-radiomics CBCT data for predicting acute toxicity following prostate cancer radiotherapy^28^. Notably, their study population included patients with gold fiducials, introducing significant artefact-related changes in imaging parameters and representing a key limitation. Additionally, their analysis pooled patients across varying radiotherapy treatment concepts, potentially obscuring treatment-specific effects. Bosetti et al. evaluated the weekly progression of CBCT-based radiomic features during normofractionated radiotherapy for prostate cancer, demonstrating the predictive potential of histogram and shape based radiomic features for biochemical recurrence^27^. Similarly, our findings revealed an overall decline in the stability of shape-based radiomic features and a progressive reduction in stability within the target structure, the prostate, with increasing radiotherapy fractions. However, as our cohort underwent ultra-hypofractionated adaptive SBRT, further research is necessary to determine whether these predictive potentials can be effectively translated to this high-precision radiotherapy concept.

Unlike previous studies, our trial investigated radiomic features on a fractional basis, offering a comprehensive analysis of their longitudinal course across all fractional CBCT scans acquired during aRT. Furthermore, to the best of our knowledge, this is the first study to longitudinally characterize radiomic features during adaptive SBRT for prostate cancer and to conduct radiomics analysis using the advanced HyperSight-CBCT-based scans without artefact-related compromises introduced by gold fiducials.

The integration of HyperSight-CBCT based scans, a novel imaging mode offering superior image quality, represents a paradigm shift for radiation oncology^10^. This development facilitates the application of quantitative imaging analysis to fractional imaging scans, unlocking new possibilities for clinical translation and quantitative imaging in high-precision radiotherapy. Kunnen et al. demonstrated consistently high image quality for IGRT in prostate cancer^12^, enabling the precise delineation of anatomical structures required for aRT and subsequent advanced, longitudinal radiomics analysis, as conducted for the first time by this study. Moreover, unlike the IGRT population studied by Kunnen et al., our study focused on patients treated with the aRT radiation mode, providing clinically validated, real-world organ delineation in HyperSight-CBCT-based imaging for adaptive treatment planning^12^. Furthermore, to the best of our knowledge, this is the first study to perform a pre-therapeutic robustness analysis of clinical radiomic features derived from pelvic HyperSight-CBCT scans. The observed very high robustness of radiomic features indicates that HyperSight-CBCT imaging can provide radiomic features, which could potentially serve as reliable radiological markers, forming a foundation for the clinical application of radiomics. Although this analysis makes the bias of contouring and test–retest variability in the presented results less likely, such a relevant factor needs to be further considered and re-evaluated, especially in studies on advanced imaging in high-precision aRT.

The detailed analysis of the longitudinal course of fractional radiomic features in this study sets a new benchmark for quantitative imaging in high-precision radiotherapy and aims to serve as a template for future trials exploring the clinical role of HyperSight-CBCT-based imaging. Such trials could investigate both early treatment response, enabling response-specific adaptive treatment strategies, and the monitoring of normal tissue changes throughout the radiotherapy treatment course. This approach might support localized treatment intensifications, correspondingly to MR-based dose escalations in intraprostatic high risk areas^40,41^. With multiparametric MRI already providing promising targets for treatment intensification^40,41^ and quantitative MRI during RT offering radiological biomarkers for treatment response^42,43^, the novel quantitative HyperSight-CBCT imaging could similarly establish a CT-based signature of prostate cancer response to high-precision RT, potentially reducing dependency on additional MRI examinations. This innovation could advance personalized radiotherapy concepts, especially when combined with adaptive RT and artificial intelligence for early response prediction^44,45^ and toxicity monitoring^46^. Incorporating fractional, quantitative HyperSight-CBCT imaging and its radiomic features into clinical workflows, particularly for tissue change assessments, might improve the effectiveness of clinical observation and dose constraints, thereby facilitating more comprehensive patient monitoring.

This study has several limitations. Conducted in a monocentric population of 32 patients, the findings require further validation. However, the clinical dataset comprising 160 fractional CBCT scans is characterized by a homogeneous cohort of images that have been directly segmented for clinical use in high-precision aRT. The results demonstrated here are specific to the CBCT presets ‘pelvis’ and ‘pelvis MAR,’ requiring further exploration of other available presets and their respective topographic scan regions or tumor sites. Additionally, the HyperSight-CBCT-based scans were performed using iCBCT as the reconstruction mode, which is characterized by a short reconstruction time. Thus, validation with the Acuros reconstruction mode of HyperSight-CBCT scans remains essential. The radiomics course analyzed during high-precision adaptive SBRT, incorporating varying target delineation concepts depending on the risk stage, also warrants evaluation for its applicability to alternative dose and delineation concepts. Furthermore, translating these findings to standard IGRT (non-adaptive radiotherapy), characterized by decreased precision in dose application, is necessary, particularly for normofractionated and moderately hypofractionated dose concepts, which remain more prevalent in prostate cancer radiotherapy. While the stability of radiomic features was assessed for selected VOIs, other vulnerable organs at risk, such as the bladder and rectum, were excluded from the analysis due to their considerable variability, particularly influenced by the filling, which compromises the robustness of the radiomic features. However, as these structures are susceptible to radiation-induced toxicity, assessing their longitudinal radiomic stability—specifically of the affected organ wall—could be a meaningful aspect of future studies. Additionally, since pre-therapeutic robustness analysis was based on the prostate, the impact of contouring variability on other VOIs remains to be determined, especially when including variable organs such as bladder or rectum in the analysis. Finally, radiomic feature extraction was conducted using a single software tool. Future studies should validate these results with alternative software and expand the analysis to include a broader spectrum of radiomic features and different extraction concepts.

## Conclusion

In summary, this study provides the first comprehensive analysis of the novel, diagnostic-like HyperSight-CBCT based imaging, focusing on the longitudinal fractional course of radiomic features during adaptive SBRT for prostate cancer. The observed trends set a new standard for advanced imaging in radiotherapy and offer a robust framework for future research in CT-guided adaptive radiotherapy. Importantly, these findings will contribute to the identification of radiological biomarkers within the context of high-precision radiotherapy. These insights are poised to drive the development of personalized treatment strategies for patients with radiotherapy for localized prostate cancer.

## Data Availability

The data used and generated in this study may be made available, subject to ethical and data protection considerations, upon reasonable request on an individual basis. Please contact Constantin Dreher, MD (E-mail: constantin.dreher@medma.uni-heidelberg.de) to request the data.
